# A case of rhinocerebral mucormycosis with brain abscess drained by endoscopic endonasal skull base surgery

**DOI:** 10.1016/j.mmcr.2020.09.004

**Published:** 2020-09-25

**Authors:** Kensuke Uraguchi, Kenichi Kozakura, Satoshi Oka, Takaya Higaki, Seiichiro Makihara, Toshi Imai, Akira Doi, Tsuyoshi Ohta, Shin Kariya, Kazunori Nishizaki

**Affiliations:** a Department of Otolaryngology-Head and Neck Surgery, Okayama University Graduate School of Medicine, Dentistry and Pharmaceutical Sciences, Okayama, Japan; b Department of Otorhinolaryngology, Kochi Health Sciences Center, Kochi, Japan; c Department of Hematology and Blood Transfusion, Kochi Health Sciences Center, Kochi, Japan; d Department of Otorhinolaryngology, Head and Neck Surgery, Kagawa Rosai Hospital, Kagawa, Japan; e Department of Neurosurgery, Kochi Health Sciences Center, Kochi, Japan

**Keywords:** Rhinocerebral mucormycosis, Acute rhinosinusitis, Brain abscess, Endoscopic endonasal skull base surgery, Acute monocytic leukemia

## Abstract

A 70-year-old Japanese man undergoing remission induction therapy for acute monocytic leukemia (AML-M5b) developed fever and headache, and was started on antibiotics and liposomal amphotericin B (L-AMB). There was no improvement, and computed tomography and contrast-enhanced magnetic resonance imaging revealed acute rhinosinusitis and brain abscess. Successful endoscopic endonasal surgery was performed at this point, providing drainage for the rhinosinusitis and abscess. Histopathological findings showed the mucormycosis.

## 1 Introduction

Rhinocerebral mucormycosis is a rare but life-threatening fungal infection that usually occurs in immunocompromised patients [1], and brain abscess could develop from the sinus infection [2]. Rhinocerebral mucormycosis in the paranasal sinus has been treated by aggressive surgical debridement and antifungal drug therapy; however, it is difficult to decide the appropriate surgery for brain abscesses [[3], [4], [5], [6]]. Here, we report a case of endoscopic endonasal skull base surgery performed for rhinocerebral mucormycosis with brain abscess.

### 1.1 Case presentation

A 70-year-old Japanese man with general malaise and pancytopenia was referred to the hematology department for evaluation. He had a past history of untreated diabetes and chronic renal disease. He was diagnosed with acute monocytic leukemia (AML-M5b) by bone marrow aspiration, and the cerebrospinal fluid (CSF) cytology was Class III. The patient was started on remission induction therapy with a combination of cytarabine and daunorubicin and received intrathecal chemotherapy aiming at prevention for relapse of central nervous system.

On the 16th day from start of the remission induction therapy, although we had administrated antibiotic treatment with 1.5g/day meropenem and antifungal treatment with 150 mg/day micafungin for febrile neutropenia as empiric therapy, he had fever and headache. Laboratory tests revealed a white blood cell count of 150/μl (normal range: 3500–9000), hemoglobin level of 6.9 g/dl (normal range: 13.4–17.4), platelet count of 20,000/μl (normal range: 120,000–350,000), HbA1c level of 6.5% (normal range: 4.6–6.2), creatinine clearance of 28.1 ml/min (normal range: 76.0–140.3) in a 24-h urine collection test, *C*-reactive protein level of 10.57 mg/dl (normal range: 0.00–0.30), and procalcitonin of 0.84 ng/ml (normal range: <0.50). Moreover, the results for beta-D-glucan, which was 12.4 pg/ml (normal range: 0.00–20.0), and aspergillus galactomannan antigen in serum were negative. Two sets of blood culture test were negative. Therefore, we changed antibiotic treatment to 500 mg/day levofloxacin and changed the antifungal treatment to 2.5 mg/kg/day liposomal amphotericin B (L-AMB).

**Fig. 1:**
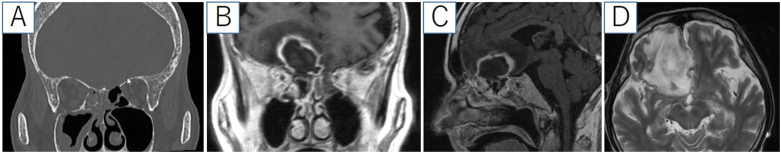
Preoperative CT and contrast-enhanced MRI: (A) CT showing acute rhinosinusitis without bone erosion; (B, C) gadolinium-contrasted T1-weighted image showing a brain abscess with a rim of peripheral enhancement localized in the frontal sinus adjoined the skull base at the posterior ethmoid sinus; (D) T2-weighted image showing brain edema in the frontal lobe around the brain abscess.

On the 22nd day from start of the remission induction therapy, there was no improvement in clinical symptoms. Computed tomography and magnetic resonance imaging (MRI) showed a soft tissue shadow in the right ethmoid and sphenoid sinus (Fig. 1A), and a slight brain edema in the frontal lobe adjoining the paranasal shadow without bone erosion of the skull base. We suspected acute rhinosinusitis gave rise to intracranial lesions, and therefore changed treatment from levofloxacin to 4 g/day meropenem while also increasing the L-AMB dosage to 5 mg/kg/day. The patient had persistent fever, intermittent headache and presented with a syndrome of inappropriate secretion of ADH (SIADH). On the contrary, the patient did not have nasal discharge, local skin ulceration and other neurologic symptoms.

On the 32nd day from start of the remission induction therapy, contrast-enhanced MRI showed a brain abscess and extension of brain edema localized in the frontal lobe adjoined the skull base at the posterior ethmoid sinus (Fig. 1B–D). An endoscopic examination of the nasal cavity revealed a normal membrane without necrotic tissue and nasal discharge. Laboratory tests revealed a white blood cell count of 4430/μl, hemoglobin level of 8.2 g/dl, platelet count of 81,000/μl, and *C*-reactive protein level of 0.52 mg/dl, and beta-D-glucan level of 12.0 pg/ml. Therefore, the patient was referred to the otolaryngology department and received emergency surgery on the same day.

**Fig. 2:**
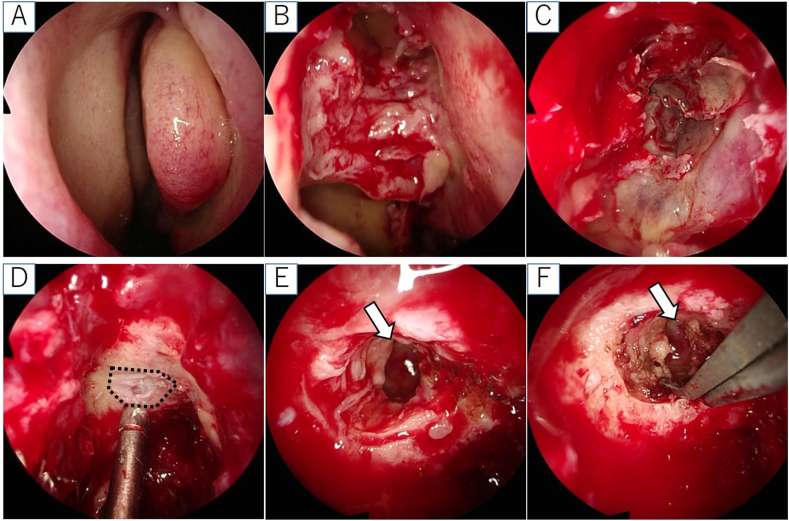
Endoscopic endonasal skull base surgery: (A, B) no necrotic membrane in the middle meatus: (C) necrotic membrane in the post ethmoidal sinus; (D) exposed dura mater on the ethmoidal roof after drilling (black dashed lines); (E) abscess cavity and slight cerebrospinal fluid leak; (F) cauterized area between the dura matter and brain abscess wall. The white arrow points to the abscess cavity.

We performed endoscopic endonasal skull base surgery in cooperation with a neurosurgeon (see in supplemental material). There was no necrotic membrane in the middle meatus and the maxillary, frontal, and anterior ethmoidal sinuses (Fig. 2A and B). The posterior ethmoidal sinus membrane appeared to be necrotic (Fig. 2C). In the sphenoid sinus, pus leaked out, and the membrane was light red without necrosis. With a navigation system, we drilled into the posterior ethmoid roof and exposed the dura mater (Fig. 2D), then resected the dura mater, and drained the brain abscess. The abscess had whitish-yellowish pus, which we washed with a saline solution (Fig. 2E). Although CSF leakage occurred, we stopped it by cauterizing the area between the dura mater and brain abscess wall (Fig. 2F). A biopsy was taken from the membrane in the anterior and posterior ethmoidal sinuses, membrane in the sphenoid sinus, dura mater and brain abscess wall. Pus from the sphenoid sinus and discharge from the brain abscess were also taken for culture tests. At the end of the intervention, we packed the nasal cavity with chitin-coated gauze.

**Fig. 3:**
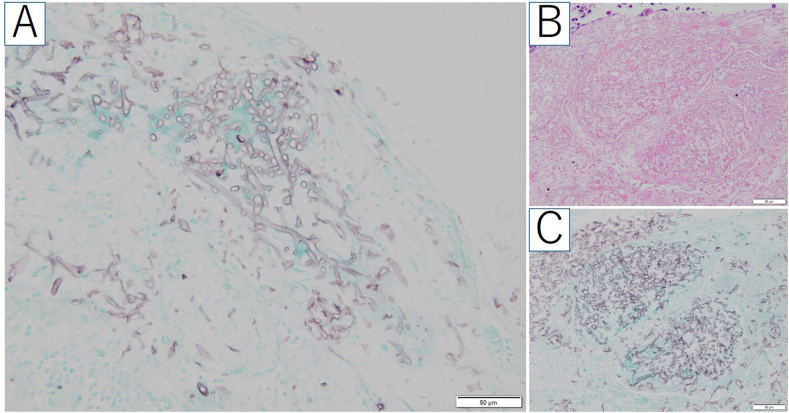
Histopathological examination: (A) hyphae branching and spreading at acute angles (Glocotto stain; scale bar = 50μm); (B,C) some hyphae invading blood vessels in the membrane (H＆E and Grocott stain; scale bar = 50μm).

After the operation, we increased the L-AMB dosage to 10 mg/kg/day on the assumption that the patient had rhinocerebral mucormycosis, considering his immunodeficiency and necrotic membrane. A histopathological examination found broad, non-septate hyphae growing in almost all of the necrotic membrane, and hyphae branching and spreading at acute angles within the posterior ethmoidal sinus membrane and brain abscess (Fig. 3A). Some hyphae invaded blood vessels in the membrane (Fig. 3B and C). The culture test results of the pus from the sphenoid sinus and brain abscess were negative. After diagnosing rhinocerebral mucormycosis with brain abscess, we suggested to the patient to use nasal irrigation with saline by himself, but he rejected this instruction. The postoperative course was uncomplicated, with neither CSF leakage nor meningitis, and he recovered from the fever and headache. On the 17th postoperative day (on the 49 day from start of the remission induction therapy), a bone marrow examination showed recurrence of AML-M5b. On the 21st postoperative day, MRI showed reduction of the brain abscess, the remaining brain edema, and air in the brain abscess. On the 30th postoperative day, the patient was started on azacytidine for the recurrence of AML-M5b. Afterward, on the 42nd postoperative day, the patient died from gram-negative septic shock due to an intestinal infection.

## 2 Discussion

The aggressive hallmarks of mucormycosis easily lead to high mortality because of delays in diagnosis and incorrect treatment. Rhinocerebral mucormycosis is the most common clinical presentation of mucormycosis, which is caused by inhalation of spores [7]. Mucormycosis can spread from the paranasal sinus to extranasal sites such as skin, orbital, and brain lesions. Although the most frequent causes of brain abscesses are *Streptococcus* and *Staphylococcus* species [8], in immunocompromised patients the differential diagnosis of causative organisms is much broader. Mucormycosis may invade the central nervous system in 13.3–22% of cases with haematological disorders, and the most common form of invasive disease is rhino-orbital-cerebral mucormycosis, reported in 33–49% of patients [2]. However, Therakathu et al. reported that brain abscess was rare, occurring in 2 out of 43 patients with rhino-orbital-cerebral mucormycosis [9]. The basic treatment for mucormycosis is a combination of aggressive surgical debridement and antifungal drug therapy such as with intravenous L-AMB [10]. Despite the aggressive surgical and antifungal drug treatment, mucormycosis has been a life-threating disease with a poorer prognosis than viral and bacterial infections [4]. Furthermore, surgery is sometimes impossible to perform in cases of disseminated mucormycosis or infected organs that are difficult to reach, such as certain parts of the brain [5].

In this reported case, because the brain abscess developed from acute rhinosinusitis in spite of antibiotic and antifungal therapy. The decision to perform the surgery came after discussions with a neurosurgeon, hematologist, and anesthetist. Although neurosurgery offered several more common approaches such as craniotomy and stereotactic aspiration, we chose endoscopic endonasal skull base surgery for two reasons. First, the immunosuppressed patient was in poor general condition, so he could not tolerate craniotomy, and stereotactic aspiration could penetrate the abscess and lead to meningitis. Second, debridement was required for the sinusitis, which progressed to brain abscess by vascular invasion penetrating the skull base. The surgery was also expected to be easier to perform than other approaches because the brain abscess adjoined the skull base at the posterior ethmoid sinus. For these reasons, an endonasal approach was suitable for diagnosis and provided a less traumatic option [10].

Preoperatively, we planned to use a mucosal flap, fascia, and fat to seal any CSF leak that occurred, but also expected difficulties in using them for reconstruction because the packing for the brain abscess might be inappropriate and the mucosal flap might lead to necrosis by infection. If the vascularized mucosal flap was unsuccessful to seal CSF leak, we planned to use abdominal fat filling into cavity as plug and nasal packing for general compression temporarily. Actually, there was a small CSF leak from a crack between the dura mater and brain abscess wall during the operation, but we unexpectedly were able to stop the leak by cauterizing the crack and agglutinating cells.

A few cases of brain abscess drained by endoscopic endonasal skull base surgery have been reported [11,12]. In the paper on invasive fungal sinusitis with brain abscess, a similar case caused by *Aspergillus* spp was discussed; the brain abscess that had spread from the sinusitis was drained by an endonasal *trans*-ethmoidal approach, though there was no CSF leak and the aperture was left open for drainage [11]. As for mucormycosis, there were a few case reports of the skull base mucormycosis without brain abscess resected by endoscopic endonasal skull base surgery. In these reports, mucormycosis spread to neighboring structure with bone erosion [13,14]. To our knowledge, this paper presents the first report of rhinocerebral mucormycosis with brain abscess drained by endoscopic endonasal skull base surgery. In this case, brain abscess might have been developed from acute rhinosinusitis through vascular invasion penetrating the skull base without bone erosion. The advantage of this endonasal endoscopic surgery for brain abscess is that the procedure is minimally invasive and relatively simple to perform, so the surgery could be considered as a possible treatment for brain abscess.

## Funding source

This work was supported by 10.13039/501100001691JSPS KAKENHI (Grants in aid for scientific research). Grant Number JP17K11329.

## Consent

Written informed consent was obtained from the patient or legal guardian(s) for publication of this case report and accompanying images. A copy of the written consent is available for review by the Editor-in-Chief of this journal on request.

## Declaration of competing interest

There are none.
